# Genome-informed diagnostics for specific and rapid detection of *Pectobacterium* species using recombinase polymerase amplification coupled with a lateral flow device

**DOI:** 10.1038/s41598-018-34275-0

**Published:** 2018-10-29

**Authors:** Firas A. Ahmed, Adriana Larrea-Sarmiento, Anne M. Alvarez, Mohammad Arif

**Affiliations:** 1 Department of Plant and Environmental Protection Sciences, University of Hawaii at Manoa, Honolulu, HI United States; 2 Agriculture College, University of Kufa, Al-Najaf, Iraq

## Abstract

*Pectobacterium* species cause serious bacterial soft rot diseases worldwide on economically important fruit and vegetable crops including tomato and potato. Accurate and simple methods are essential for rapid pathogen identification and timely management of the diseases. Recombinase polymerase amplification (RPA) combined with a lateral flow device (LFD) was developed for specific detection of *Pectobacterium* sp. directly from infected plant materials with no need for DNA isolation. The specificity of RPA-LFD was tested with 26 *Pectobacterium* sp. strains and 12 non-*Pectobacterium* species and no false positive or false negative outcomes were observed. RPA primers and probe for host control were also developed to detect the host genome for enhanced reliability and accuracy of the developed assay. The detection limit of 10 fg was obtained with both sensitivity and spiked sensitivity assays. No inhibitory effects were observed on the RPA assay when targets (pathogen and host) were directly detected from infected potato and tomato sap. The developed RPA assay has numerous applications from routine diagnostics at point-of-care, biosecurity, surveillance and disease management to epidemiological studies. In addition, this tool can also be used to discover reservoir hosts for *Pectobacterium* species.

## Introduction

Soft rot bacteria in the genus *Pectobacterium* (formerly *Erwinia* in the family *Enterobacteriaceae*) affect a wide range of host plants worldwide and cause significant economic losses in the field, storage and during transit^1^. *Pectobacterium* comprises different species that cause wilts, stem rots, soft rots and postharvest diseases on many fruit and vegetable crops. Several devastating species including *P*. *carotovorum*, *P*. *atrosepticum*, *P*. *betavasculorum* and *P*. *wasabiae* affect both monocot and dicot hosts^2,3^. Both, *Pectobacterium* sp. and *Dickeya* sp. (also in the family *Enterobacteriaceae*) share a common host range that includes potato, tomato, pepper, tobacco, broccoli^4,5^. Kim and coworkers^6^ demonstrated that these pathogens exist as mixed populations in infected plant tissue. Discrimination of *Pectobacterium* and *Dickeya* in plant tissues is challenging because pathogens in these genera produce similar symptoms on similar hosts. Rapid diagnostic tools that can differentiate *Pectobacterium* from *Dickeya* and closely related genera in the *Enterobacteriaceae* are needed to follow the epidemiology of these diseases and to determine sources of contamination.

Traditional bacteriological practices are time-consuming, labor intensive, and require trained personnel to distinguish genera consistently. The polymerase chain reaction (PCR) has become the most widely used method for accurate detection of plant pathogens^7^. However, PCR-based methods have some disadvantages - they are time-consuming, require a sophisticated and expensive thermal cycler, and cannot conveniently be used at point-of-care^8^. Several isothermal methods based on different principles and chemistries are now available^9^ and can be performed at point-of-care without the need of an expensive thermal cycler. These include: strand displacement amplification (SDA)^10^, loop-mediated isothermal amplification (LAMP)^11^, rolling circle amplification (RCA)^12^, helicase-dependent amplification (HDA)^13^, recombinase polymerase amplification (RPA)^14^ and nicking enzyme amplification reaction (NEAR)^15^. Yasuhara‐Bell and coworkers^16^ (2016) reported the development of a specific loop-mediated isothermal amplification (LAMP) assay to detect the blackleg pathogen, *P*. *atrosepticum* and soft rot pathogen, *P*. *carotovorum*. However, LAMP has disadvantages including complexity in primer design, high temperature requirement (65 °C) and a relatively expensive portable device for field assays. In recent years, RPA has received much attention because of its insensitivity to inhibitors, eliminating the need for DNA isolation. RPA also has a comparatively low temperature requirement (37 °C to 42 °C) and freeze-dried TwistDx RPA reagents are commercially available. This is a highly sensitive, rapid and accurate isothermal detection method that can be performed at the point-of-care with minimum requirements^17^. Nevertheless, false negative results can also occur through errors in DNA preparation and/or inhibitors; thus, an internal/host control is needed to validate the successful run of each reaction and enhance the reliability of the test^18,19^.

The objective of this study was to develop an RPA-LFD assay for specific and rapid detection of *Pectobacterium*. The developed assays are rapid and can reliably detect the target pathogen at point-of-care with a minimal requirement for laboratory equipment. The assay has applications in routine diagnostic surveys for biosecurity, disease management and disease epidemiology.

## Results

### Target selection *in-silico* validation

**Figure 1:**
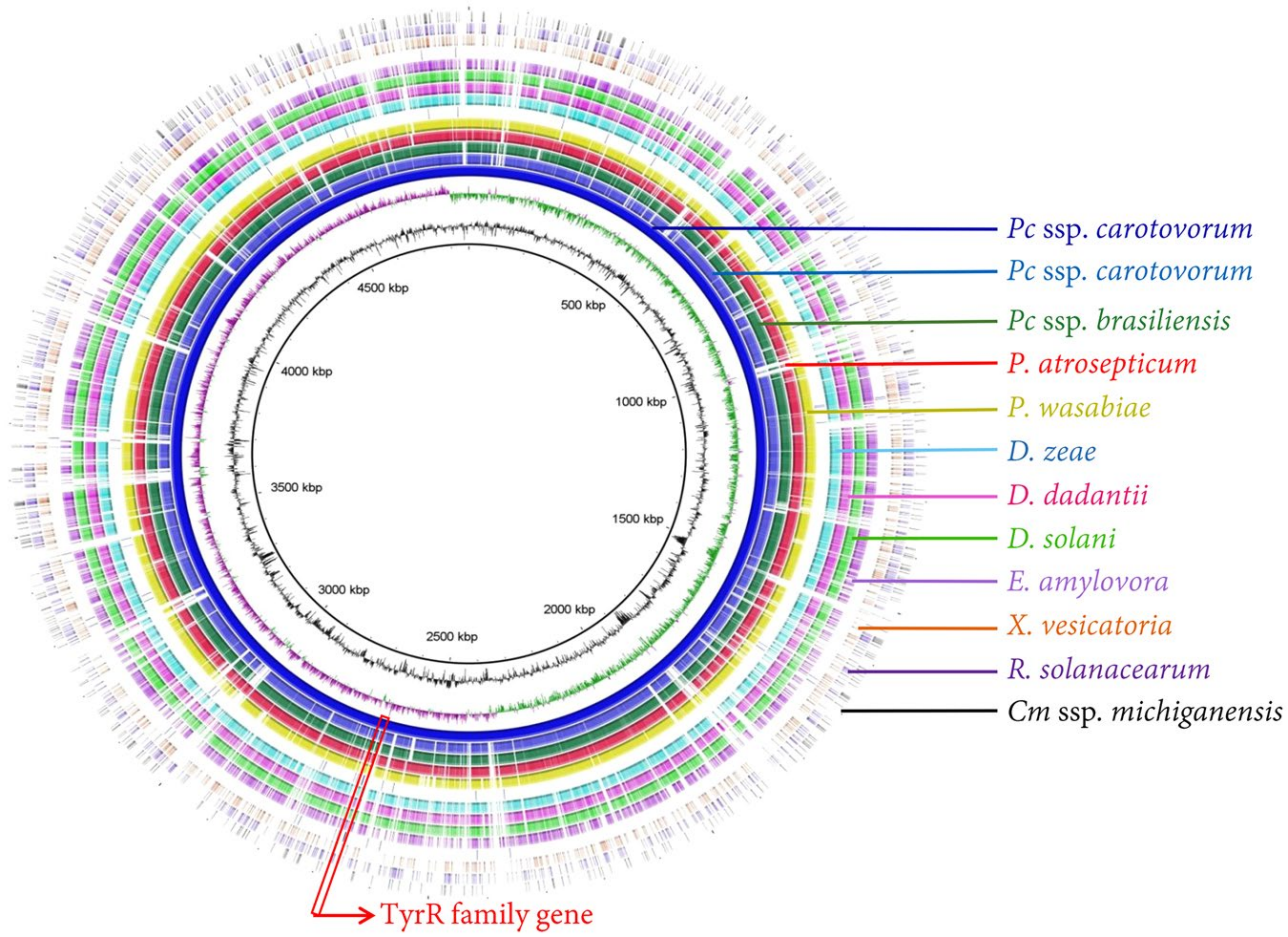
Ring image was generated to show the location of *tyrR* gene and genomic variation among the genomes. All genomes were retrieved from NCBI GenBank genome database. Gene *tyrR* was used to design recombinase polymerase amplification (RPA) primers and probe for specific detection of the genus *Pectobacterium*. The genome ring image from the inside out shows: genome coordinates (kbp), GC content (black), GC skew (purple/green). Other rings show BLASTn comparisons of 12 complete genomes as labelled. *Pectobacterium carotovorum* subsp. *carotovorum* (NCBI accession number NC_012917) was used to generate the ring image using BRIGS. Other genomes and their NCBI accession numbers: *Pectobacterium carotovorum* subsp. *carotovorum* (NC_18525), *Pectobacterium carotovorum* subsp. *brasiliensis* (NZ_CP020350), *P*. *atrosepticum* (NZ_CP009125), *P*. *wasabiae* (NC_013421)*; Dickeya zeae* (NZ_CP006929) *D*. *dadantii* (NC_014500), *D*. *solani* (NZ_CP009460), *Erwinia amylovora* (NC_013961), *Xanthomonas vesicatoria* (NZ_CP018470), *Ralstonia solanacearum* (NC_003295) and *Clavibacter michiganensis* subsp. *michiganensis* (NC_009480). Plasmid sequences were not included in the analysis.

**Figure 2:**
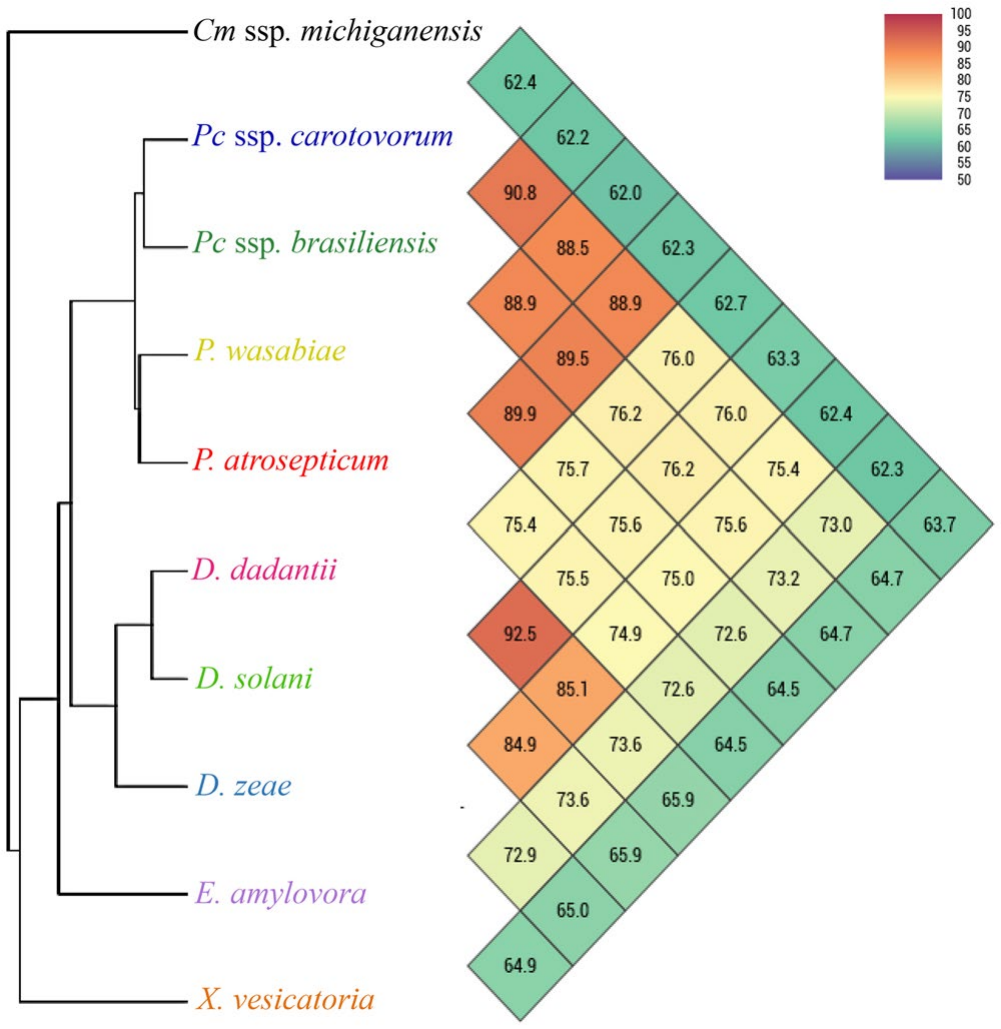
Overall orthologous average nucleotide identity (ANI) among bacterial genomes was calculated using Orthologous Average Nucleotide Identity Tool version 0.93 (OrthoANI). Values in color matrix boxes indicate the similarity percentage among the genomes. *Clavibacter michiganensis* subsp. *michiganensis* (NCBI accession number NC_009480), *Pectobacterium carotovorum* subsp. *carotovorum* (NCBI accession number NC_012917), *P*. *carotovorum* subsp. *brasiliensis* (NCBI accession number NZ_CP020350), *P*. *wasabiae* (NCBI accession number NC_013421), *P*. *atrosepticum* (NCBI accession number NZ_CP009125), *Dickeya dadantii* (NCBI accession number NC_014500), *D*. *solani* (NCBI accession number NZ_CP009460), *D*. *zeae* (NCBI accession number NZ_CP006929), *Erwinia amylovora* (NCBI accession number NC_013961) and *Xanthomonas vesicatoria* (NCBI accession number NZ_CP018470). Plasmid sequences were not included in the analysis.

Whole genome sequences of *Pectobacterium* and other genera downloaded from NCBI GenBank genome database and were explored for unique and conserved target selection of *Pectobacterium*. The *P*. *carotovorum* genome accession number NC_012798 was used as a reference for whole genome alignment and analysis. TyrR family transcription regulator gene (*tyrR)* was selected and targeted for RPA primers and probe design. Designed primers and probe were blasted against the NCBI GenBank database for *in-silico* validation. No similar sequences occurred with other genera including *Dickeya*. The primers and probe location in the genome was completed and visualized in ring image (Fig. 1). The ring image output shows a comparison of a reference genome sequence of *P*. *carotovorum* colored with dark blue in the center circle (inner most) with other closely and distantly related bacterial genomes, *P*. *carotovorum* subsp. *carotovorum* (NC_18525), *P*. *carotovorum* subsp. *brasiliensis* (NZ_CP020350) *P*. *atrosepticum* (NZ_CP009125), *P*. *wasabiae* (NC_013421), *D*. *zeae* (NZ_CP006929), *D*. *dadantii* (NC_014500), *D*. *solani* (NZ_CP009460), *E*. *amylovora* (NC_013961), *X*. *vesicatoria* (NZ_CP018470), *R*. *solanacearum* (NC_003295), and *Clavibacter michiganensis* subsp. *michiganensis* (NC_009480) (Fig. 1). The orthologous average nucleotide tool predicted overall similarity among all tested bacterial genera. Within species of *Pectobacterium*, 88.5–90.8% similarity was calculated whereas the closely related genus *Dickeya* showed 74.9–76.2% similarity with *Pectobacterium* sp. (Fig. 2). A low nucleotide similarity was observed with *E*. *amylovora*, *X*. *vesicatoria* and *C*. *michiganensis* subsp. *michiganensis* (Fig. 2). Plasmid sequences were not included in these analyses.

### Identity confirmation and phylogenetic relationships

**Figure 3:**
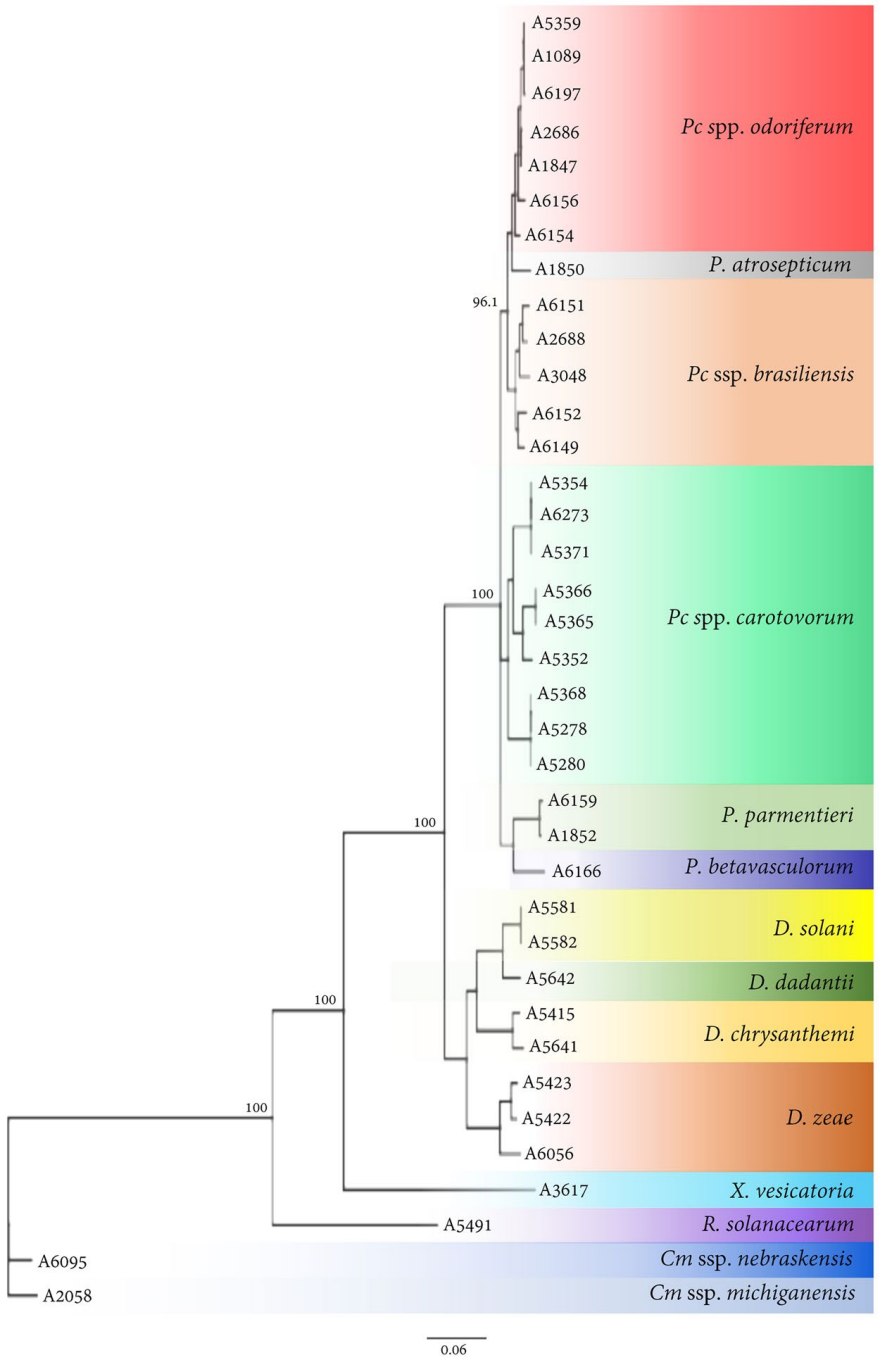
Phylogenetic analysis. Phylogenetic analysis of strains included in validation of recombinase polymerase amplification (RPA) for specific detection of *Pectobacterium* species. Consensus sequences of *dnaA* gene were aligned and used to calculate the phylogenetic relationships among the strains using NJ tree building method. Consensus tree was generated using Bootstrap resampling method with 1000 replicates. Details of the strains are given in Table 1.

**Figure 4:**
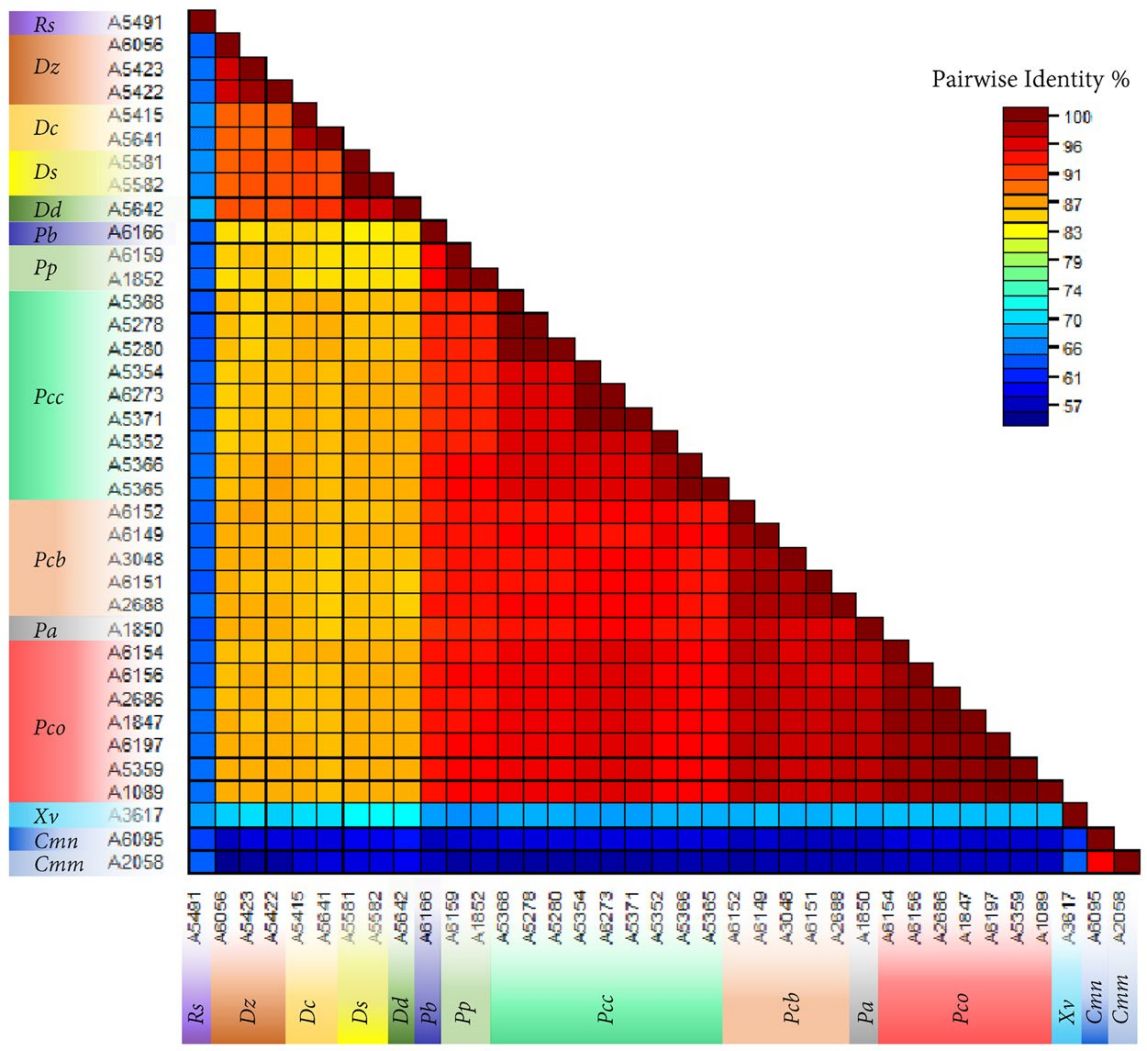
Consensus sequences of *dnaA* gene were used to generate pairwise color-code similarity matrix. All the strains used in inclusivity and exclusivity panels were included in these analyses; strain descriptions are shown in Table 1. The Sequence Demarcation Tool v1.2 was used to calculate percent pairwise similarity. *Rs – Ralstonia solanacearum; Dz – Dickeya zeae; Dc – D*. *chrysanthem*i; *Ds – D*. *solani; Dd – D*. *dadantii; Pb – Pectobacterium betavasculorum; Pp – P*. *parmentieri; Pcc – P*. *carotovorum* subsp. *carotovorum; Pcb - P*. *carotovorum* subsp. *brasiliensis; Pa – P*. *atrosepticum; Pco - P*. *carotovorum* subsp. o*doriferum; Xv – Xanthomonas vesicatoria; Cmm – Clavibacter michiganensis* subsp. *michiganensis; Cmn - C*. *michiganensis* subsp. *nebraskensis*.

All strains included in inclusivity and exclusivity panels were confirmed by amplifying partial *dnaA* gene regions; obtained sequences were blasted against NCBI GenBank nucleotide and genome databases. Sequences were also aligned, and a phylogenetic tree was generated (Fig. 3). Separate clusters were formed which further cross confirmed the BLASTn outcome. Two major clusters of *Pectobacterium* sp. and *Dickeya* sp. were formed. These two major clusters further sub-clustered according to their species or subspecies (Fig. 3). The *C*. *michiganenis* subsp. *michiganensis* and *C*. *michiganensis* subsp. *nebraskensis* showed the maximum dissimilarity with the other strains included in the analyses. Details of identity percentages among the strains are depicted in Fig. 4.

### Specificity assays

**Figure 5:**
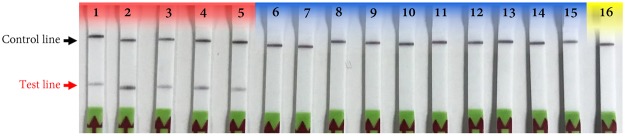
The analytical specificity of recombinase polymerase amplification (RPA) assay for target pathogen, *Pectobacterium* sp. Lane 1 - *P*. *carotovorum* subsp. *carotovorum* (A5280); lane 2 - *P*. *carotovorum* subsp. *odoriferum* (A1089); lane 3 - *P*. *carotovorum* subsp. *brasiliensis* (A2688); lane 4 - *P*. *atrosepticum* (A1850); lane 5 - *P*. *parmentieri* (A1852); lane 6 - *Dickeya zeae* (A6056); lane 7 – *D*. *zeae* (A5422); lane 8 - *D*. *dadantii* (A5642); lane 9 - *D*. *chrysanthemi* (A5641); lane 10 - *D*. *solani* (A5581); lane 11 - *D*. *solani* (A5582); lane 12 - *Clavibacter michiganensis* subsp. *michiganensis* (A2058); lane 13 - *C*. *michiganensis* subsp. *nebraskensis* (A6095); lane 14 - *Xanthomonas vesicatoria* (A3617); lane 15 - *Ralstonia solanacearum* (A5491); and lane 16 – non-template control (water).

The broad range detection capabilities of the RPA assay for *Pectobacterium* was assessed with 26 different strains of five *Pectobacterium* species/subspecies (*P*. *carotovorum* subsp. *carotovorum*, *P*. *carotovorum* subsp. *odoriferum*, *P*. *carotovorum* subsp. *brasiliensis*, *P*. *atrosepticum*, *P*. *parmentieri* and *P*. *betavasculorum*) collected from different geographical locations, whereas, the exclusivity panel included the strains from different species (*C*. *michiganensis* subsp. *michiganensis*, *C*. *michiganensis* subsp. *nebraskensis*, *D*. *zeae*, *D*. *dadantii*, *D*. *solani*, *R*. *solanacearum*, and *X*. *vesicatoria*). No false negatives or false positives were observed on the lateral flow device. Primers and probes specifically detected only *Pectobacterium* strains (Fig. 5), indicating that the developed RPA assay is accurate, robust and specific for detection of *Pectobacterium*. The summary of the specificity results is shown in Table 1.

**Table 1:** Bacterial strains used to validate the Recombinase Polymerase Amplification (RPA) method for specific detection of soft rot-causing plant bacteria in the genus *Pectobacterium*.

Lab ID	Original ID	Organism	Location	Year	Host	GenBank Accession Number	RPA Results
A5280	1-#31	*Pectobacterium carotovorum* subsp. *carotovorum*	Hawaii	2004	Irrigation water	MH453512	+
A5278	1-#21	*P*. *carotovorum* subsp. *carotovorum*	Hawaii	2004	Irrigation water	MH453511	+
A5368	5X	*P*. *carotovorum* subsp. *carotovorum*	Hawaii	1994	*Aglaonema* sp.	MH453510	+
A5366	3 C	*P*. *carotovorum* subsp. *carotovorum*	Hawaii	1994	*Aglaonema* sp.	MH453530	+
A5371	CC26	*P*. *carotovorum* subsp. *carotovorum*	Hawaii	1994	*Aglaonema* sp.	MH453528	+
A5349	5 C	*P*. *carotovorum* subsp. *carotovorum*	Hawaii	1994	*Aglaonema* sp.	*	+
A5352	T-15	*P*. *carotovorum* subsp. *carotovorum*	Hawaii	1994	*Aglaonema* sp.	MH453529	+
A5365	2B	*P*. *carotovorum* subsp. *carotovorum*	Hawaii	1994	*Aglaonema* sp.	MH453531	+
A6273	BA17	*P*. *carotovorum* subsp. *carotovorum*	Hawaii	2015	Tomato	MH453527	+
A5354	11X	*P*. *carotovorum* subsp. *carotovorum*	Hawaii	1994	*Aglaonema* sp.	MH453526	+
A1089	QR-11	*P*. *carotovorum* subsp. *odoriferum*	California	1977	Pepper	MH453518	+
A1847	IPM 60	*P*. *carotovorum* subsp. *odoriferum*^a^	Colorado	1987	Potato	MH453520	+
A2686	E43	*P*. *carotovorum* subsp. *odoriferum*	Hawaii	1990	Cabbage	MH453519	+
A5359	EC	*P*. *carotovorum* subsp. *odoriferum*	Hawaii	2005	Papaya	MH453517	+
A6156	WPP16	*P*. *carotovorum* subsp. *odoriferum*^b^	Wisconsin	2001	Potato	MH453515	+
A6154	WPP12	*P*. *carotovorum* subsp. *odoriferum*^b^	Wisconsin	2001	Potato	MH453514	+
A6197	ER1	*P*. *carotovorum* subsp. *odoriferum*	Hawaii	1981	Cabbage	MH453516	+
A6149	WPP5	*P*. *carotovorum* subsp. *brasiliensis*	Wisconsin	2001	Potato	MH453522	+
A3048	E60	*P*. *carotovorum* subsp. *brasiliensis*	Hawaii	1991	Cabbage	MH453523	+
A2688	E45	*P*. *carotovorum* subsp. *brasiliensis*	Hawaii	1990	Cabbage	MH453525	+
A6151	WPP20	*P*. *carotovorum* subsp. *brasiliensis*	Wisconsin	2001	Potato	MH453524	+
A6152	WPP165	*P*. *carotovorum* subsp. *brasiliensis*	Wisconsin	2004	Potato	MH453521	+
A1850	IPM 1260	*P*. *atrosepticum*^c^	Colorado	1987	Potato	MH453513	+
A1852	M784	*P*. *parmentieri*	Colorado	1987	Potato	MH453534	+
A6159	WPP168	*P*. *parmentieri*	Wisconsin	2004	Potato	MH453533	+
A6166	Ecb2	*P*. *betavasculorum*	California	**	Beta vulgaris	MH453532	+
A6056	3-leaf	*Dickeya zeae*	Hawaii	2012	Pineapple	MH453535	−
A5422	CFBP2052	*D*. *zeae*	USA	1970	Corn	MH453537	−
A5423	CFBP6466	*D*. *zeae*	Martinique	1991	Pineapple	MH453536	−
A5642	CFBP 3855	*D*. *dadantii*	France	1996	*Saintpaulia*	MH453542	−
A5641	CFBP 1270	*D*. *chrysanthemi*	Denmark	1970	*Parthenium*	MH453539	−
A5415	CFBP2048	*D*. *chrysanthemi*	USA	1956	*Chrysanthemum*	MH453538	−
A5582	PRI 2188	*D*. *solani*	Israel	2007	Potato	MH453541	−
A5581	PRI 2187	*D*. *solani*	Israel	2007	Potato	MH453540	−
A2058	H-160	*Clavibacter michiganensis* subsp. *michiganensis*	Idaho	1987	Tomato	MH453508	−
A6095	20037	*C*. *michiganensis* subsp. *nebraskensis*	Nebraska	2013	Corn	MH453507	−
A3617	Xv145	*Xanthomonas vesicatoria*	South America	1990	Tomato	MH453506	−
A5491	EB2	*Ralstonia solanacearum*	Indonesia	2005	Tomato	MH453509	−

^+^Positive result; ^−^negative result; ^*^sequencing quality was not high, therefore, the sequence was not submitted to NCBI GenBank; ^**^year of isolation is not known; ^a^originally (1987) identified as *Erwinia carotovora* subsp. *carotovora;* ^b^originally (2001) identified as *Pectobacterium carotovorum* subsp. *carotovorum;* ^c^originally (1987) identified as *Erwinia carotovora* subsp. *atroseptica*. Year represents either year of isolation or year of receiving the strain in our lab.

### Sensitivity assays

**Figure 6:**
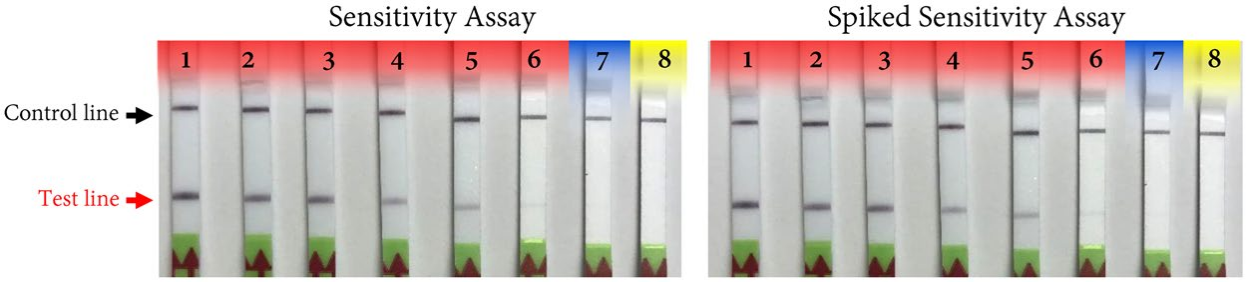
Sensitivity and spiked sensitivity of the developed RPA assay using a 10-fold serially diluted genomic DNA of *Pectobacterium carotovorum* subsp. *carotovorum* (strain A5280). In the spiked sensitivity assay, 1 $\mu {\rm{l}}$ (59 ng/ul) of host genomic DNA was added to each serially diluted genomic DNA sample to confirm the lack of an inhibitory effect of host genomic DNA on the RPA assay detection limit. No difference in sensitivity between spiked and non-spiked samples was observed. Lanes 1 to 7 are serially diluted genomic DNA (1 ng to 1 fg), and line 8 is non-template control (water).

Serially diluted (1 ng to 1 fg) genomic DNA of *P*. *carotovorum* subsp. *carotovorum* (A5280) was used to perform the sensitivity assay. The spiked assay was performed by adding 1 µl (59 ng) of healthy tomato host DNA to each reaction to confirm the inhibitory effect of host genomic DNA on RPA assay’s detection limit. Both sensitivity and spiked sensitivity assays detected *P*. *carotovorum* subsp. *carotovorum* genomic DNA up to 10 fg (Fig. 6). The outcome sensitivity detection limit was not affected when 1 µl (59 ng) of tomato host DNA was added to each reaction (Fig. 6). Non-template control was included to eliminate the possibility of getting a false positive due to cross contamination.

### Host control

RPA primers and probe were also designed targeting genomic DNA of hosts (tomato, potato, pepper and eggplant) to enhance the reliability and accuracy of the developed assays. These RPA primers and probe detected the genomic DNA of tomato fruit, tomato leaves, potato tuber, fruit of pepper and eggplant, and were used as host control in separate reaction to monitor each reaction for reliable sample/DNA prep and reagent acceptability. Each run included a positive control and a non-template control. The host control primers and probe reacted with the tested *Solanum* hosts and were detected on lateral flow strips.

### Detection of *Pectobacterium* sp. in infected host tissue

**Figure 7:**
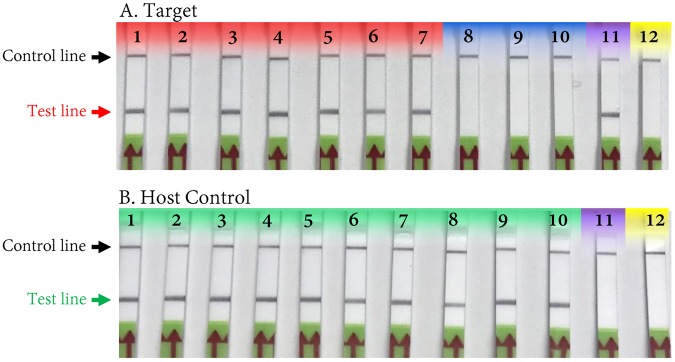
Detection of *Pectobacterium* sp. from infected plant materials using recombinase polymerase amplification (RPA). (**A**) Detection of the target pathogen (*Pectobacterium* sp.) using *Pectobacterium*-specific RPA assay; (**B**) detection of host DNA using host specific RPA assay as a host control. Lane 1 - *Pectobacterium carotovorum* subsp. *carotovorum* (A5280); lane 2 - *P*. *carotovorum* subsp. *carotovorum* (A5278); lane 3 - *P*. *carotovorum* subsp. *carotovorum* (A5368); lane 4 - *P*. *carotovorum* subsp. *brasiliensi*s (A2688); lane 5 - *P*. *carotovorum* subsp. *brasiliensis* (A3048); lane 6 - *P*. *carotovorum* subsp. *carotovorum* (A5278); lane 7 - *P*. *carotovorum* subsp. *carotovorum* (A5368); lane 8 - *Dickeya chrysanthemi* (A5415); lane 9 - *D*. *solani* (A5581); lane 10 – *D*. *solani* (A5582): lane 11 - *P*. *carotovorum* subsp. *carotovorum* (A5280; positive control – DNA from pure culture); 12) non-template control (NTC; water). Only DNAs of *Pectobacterium* sp. reacted postively and produced a line with the target specific RPA assay. Samples 1–5 were tomato inoculated with *Pectobacterium* sp., samples 6–7 were potato tubers inoculated with *Pectobacterium* sp. and samples 8–10 were potato tubers inoculated with *Dickeya* sp. Color codes: red – *Pectobacterium* sp. detected; blue – no detection; purple – positive control for *Pectobacterium* specific RPA assay but it is a negative control for host-specific RPA; green – host DNA detection; yellow – NTC.

The specificity and detection capabilities of the developed RPA assay was evaluated with infected tomato fruits and potato tubers. All tomatoes were inoculated with five different strains of *P*. *carotovorum* and three potato tubers were inoculated with two of the *P*. *carotovorum* strains. Three other potatoes were individually inoculated with a strain of *D*. *chrysanthemi* or *D*. *solani*. Positive results were obtained only from tissues of tomato and potato infected with *P*. *carotovorum* whereas no test line was observed with potato tissues infected with either of the *Dickeya* species (Fig. 7A). These results confirmed that the developed RPA assay can accurately detect the *Pectobacterium* sp. from infected plant materials. Each infected sample was also tested with host RPA primers and probe as a host control to confirm the accuracy of DNA isolation. All showed positive results with host DNA isolated from infected tissues. No amplification occurred on the non-template control (Fig. 7B).

### Rapid sample prep with TE buffer for RPA detection

**Figure 8:**
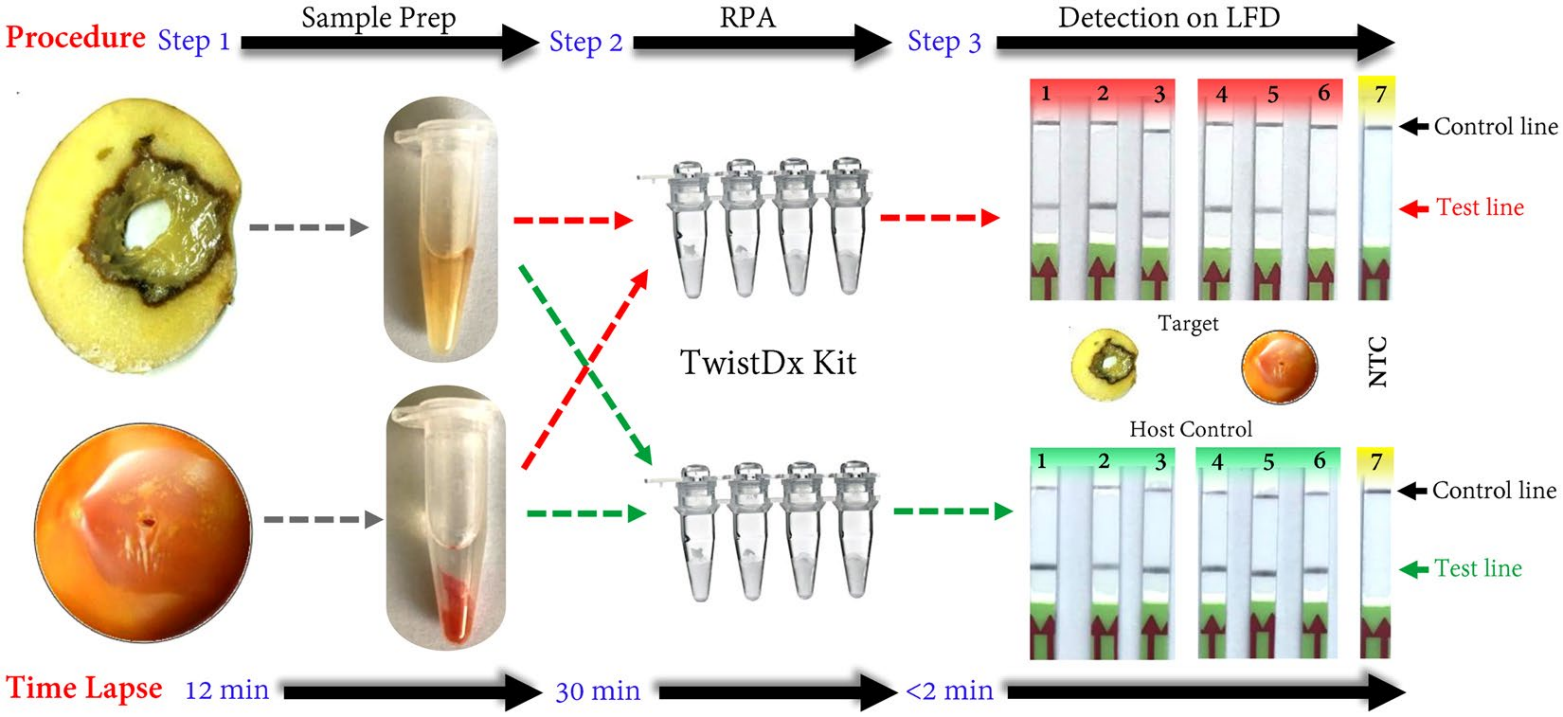
Schematic representation of direct detection of *Pectobacterium* sp. in infected host tissue using RPA-LFD and rapid sample prep with TE buffer. The results showed no inhibitory effect of plant inhibitors on RPA assay. The rapid sample prep using TE buffer takes about 12 mins. Each inoculated sample was tested with both *Pectobacterium* sp. specific RPA (upper; target) and host specific RPA (lower; host control). Color codes: red – detection of *Pectobacterium* sp. from *Pectobacterium* sp. infected potato tubers and tomato fruits; green – detection of host genome; yellow non-template control (NTC; water). Lanes 1–3 are potato tubers infected with *Pectobacterium* sp.; lanes 4–6 are tomato fruits infected with *Pectobacterium* sp.; lane 7 is NTC.

The rapid sample prep using TE buffer was evaluated with infected tomato fruits and potato tubers. Three tomato fruits and three potato tubers were artificially inoculated with *P*. *carotovorum* subsp. *carotovorum* and samples were prepared for RPA detection using TE buffer. Positive detection was obtained from infected tissues of tomato and potato with *P*. *carotovorum* subsp. *carotovorum* whereas no test line was observed in the non-template control (Fig. 8). Each infected sample was further tested with host RPA primers and probe as a host control to confirm the accuracy and the capacity of the sample prep; all showed positive results. No amplification occurred on the negative control (Fig. 8). These results confirmed that RPA-LFD assay can directly be used for specific detection with no need of DNA isolation. Total assay from sample prep to LFD based detection can be performed in less than 35 minutes without the need of lab equipment (Fig. 8).

## Discussion

We developed and validated a recombinase polymerase amplification (RPA) assay to detect and discriminate *Pectobacterium* sp. from other closely related genera including *Dickeya* sp. and non related bacterial genera such as *Clavibacter*, *Xanthomona*s, and *Ralstonia*. We also developed a rapid sample prep procedure for RPA detection using TE buffer which reduced the sample prep time to less than one minute.

LAMP methods for detection of the closely related genera *Pectobacterium* and *Dickeya* have already been developed by Yasuhara-Bell and coworkers^16^, but LAMP assays have the disadvantage of requiring a high temperature for amplification (65 °C) and an expensive instrument. RPA is a comparatively new isothermal technique that can detect target DNA in 15–30 min at 37–42 °C with no requirement for sophisticated and/or expensive instruments^20^. In addition, RPA displays greater resistance to inhibitors and can be used with samples of greater complexity as compared to other isothermal methods^21,22^. RPA is less affected by inhibitors than the well known LAMP method (M. Arif, unpublished information). Twist Dx RPA reagents are available in lyophilized form; the entire kit can be easily transported to the field without the need for cold storage. Results are easily visualized and interpretation does not require specific training or instrumentation. Ouyang and coworkers^23^ developed a Razor Ex-based sensitive field detection method for the plant pathogenic bacterium, *Xylella fastidiosa* subsp. *pauca* but its use required an expensive Razor Ex instrument and training for operation. Reagent cost per reaction was high for this instrument and limited the use of this method for routine in-field diagnostics^24^.

Due to its high sensitivity, robustness and accuracy, RPA methods using a lateral flow device are an excellent option for both in lab and out of lab detection of *Pectobacterium*. Accurate and rapid detection and identification of disease-causing bacteria are one of the most significant prerequisite aspects of disease control^25^. The successful application of RPA assay for detection of *Pectobacterium* sp. using a primer set and probe targeting TyrR family transcriptional regulator gene has been demonstrated in this study (Fig. 1). Specificity was confirmed using results from a wide range of strains in an inclusivity panel and strains from other both closely- and distantly-related plant pathogenic bacterial species. No false positive or false negative outcomes were observed (Fig. 5, Table 1). Moreover, no cross reactivity was observed when potato tuber tissues were infected with *Dickeya chrysanthemi* and *D*. *solani*, whereas tomato and potato tissues infected with *P*. *carotovorum* were all positive confirming the presence of *Pectobacterium* sp. (Fig. 7). In this study, the application of RPA-LFD assay for detection *Pectobacterium* from infected tomato fruit and potato tuber samples was achieved without extraction of DNA; the sample prep method required about 12 mins with no need of lab equipment (Fig. 8). These results have shown that the test is highly reliable for detecting *Pectobacterium* in the field. The short reaction time at 37 °C has several advantages, one of which is that samples can be warmed in hand without the need for sophisticated equipment. Secondly, because plant inhibitors have no effect on the amplification process, the test procedure does not require DNA extraction.

Diagnostic methods should be sufficiently sensitive to detect a low amount of the target pathogen in an infected sample without producing false negative results. The developed RPA assay is sensitive, detecting 10 fg of pure *Pectobacterium* DNA and 10 fg *Pectobacterium* DNA mixed with host DNA. The detection limit was the same for *Pectobacterium* DNA as well as bacterial DNA mixed with host DNA indicating that host DNA had no adverse impact on the assay (Fig. 6). The inclusion of a host control in the diagnostic assay targeting the host genome enhances the reliability of the outcome. The developed RPA assay also included host specific RPA primers and probe to detect the genome of potato, tomato, pepper and eggplant. The newly-developed RPA assay is ready to use for in-field and/or storage applications for accurate and robust detection of *Pectobacterium* sp. in tomato and potato.

## Conclusion

We have established a novel lateral flow strip-based RPA assay for *Pectobacterium* detection that provides unique advantages with respect to speed, specificity, sensitivity, ease of visual detection and simplicity of equipment required. The developed RPA assay does not require pure DNA isolation and a sample can be prepared for RPA in less than a minute. RPA primers and probe were developed to discriminate between host and pathogen, enhancing the reliability of the assay. All these features indicate that the RPA assay can be a useful tool for laboratory and field applications for specific detection of *Pectobacterium*.

## Materials and Methods

### Source of bacterial strains and DNA isolation

The bacterial strains used in both inclusivity and exclusivity panels are listed in Table 1. Strains were stored in −80 °C and re-grown on TZC medium (10 g/L peptone, 5 g/L glucose 17 g/L agar, and 0.001% 2, 3, 5-triphenyle-tetrazolium chloride added after autoclaving); plates were incubated at 26 °C ± 2 °C. The Wizard Genomic DNA Purification Kit (Promega, Madison, WI) was used to extract the genomic DNA from all strains according to the manufacturer’s protocols. The concentration of extracted DNA was measured using a NanoDrop^TM^ 2000C (Thermo Fisher Scientific Inc, Worcester, MA). Extracted DNA was stored in a freezer at −20 °C. For host samples, healthy peppers, eggplants, tomatoes, and potato were grown in the greenhouse. Fruits and tubers were surface sterilized with 10% sodium hypochlorite for 1 min and washed three times with sterilized water. The Wizard Genomic DNA Purification Kit was used to extract the genomic DNA from all fruit samples following the manufacturer’s protocols.

### Endpoint PCR and *dnaA* sequencing

The *dnaA* gene region of bacterial strains was amplified using primer sets designed for *Clavibacter*, *Dickeya*, *Pectobacteriu*m, *Ralstonia*, and *Xanthomonas* (Supplemental Table 1)^26^. A 20 μl PCR reaction contained 10 μl of GoTaq Green Master Mix (Promega), 0.5 μl of 10 mM of each forward and reverse primer, 1 μl template DNA, and 8 μl Ultra-Pure DNase/RNase-Free distilled water (Thermo Fisher Scientific). PCR was performed with an initial denaturation step at 95 °C for 5 minutes, followed by 35 cycles of 94 °C for 20 seconds, 60 °C for 20 seconds and 72 °C for 20 seconds, followed by a three mins extension at 72 °C and hold at 12 °C. Agarose gel (1.5%) electrophoresis was used to separate all PCR amplicons, stained with 0.4 μg/ml ethidium bromide, and bands were visualized under a UV illuminator. PCR was performed in a T100 Thermal cycler (Bio-Rad, Hercules, CA). Amplified PCR products were treated using ExoSAP-1T (Affymetrix Inc., Santa Clara, CA). A 5 μl of post-PCR reaction and 2 μl ExoSAP-IT reagents were combined and incubated at 37 °C for 15 min following by an incubation of 80 °C for 15 min. Each treated template was sequenced for both sense- and antisense-strands using corresponding genus-specific primers. Sequencing was performed at the Advanced Studies in Genomics, Proteomics and Bioinformatics facility (ASGPB), University of Hawaii at Manoa, Honolulu, HI.

### Sequence analyses

The sequences of partial *dnaA* gene regions of tested strains were manually edited in order to generate error free consensus sequences for all the strains. Manually edited error free consensus sequences were compared against the NCBI GenBank nucleotide database using BLASTn algorithm. Consensus sequences were aligned and used to reveal the phylogenetic relationships among the strains using NJ tree building method. The consensus tree was generated using the Bootstrap resampling method with 1000 replicates^27^. Geneious 10.2.3 was used for editing, alignment, and generation of phylogenetic trees. The Sequence Demarcation Tool v1.2 was used to calculate percent pairwise similarity among the strains.

### *Pectobacterium* genus-specific RPA primers and probe design

The genomes of *P. carotovorum* subsp. *carotovorum*, *P. carotovorum* subsp. *odoriferum*, *P. atrosepticum*, *P. wasabiae*, *D. zeae*, *D. dadantii*, *D. solani*, *E. amylovora*, *C. michiganensis* subsp. *michiganensis*, *C. michiganensis* subsp. *nebraskensis*, *X. vesicatoria* and *R. solanacearum* were retrieved from NCBI GenBank genome database. Genomes were aligned using Mauve (2.4.0); and *P. carotovorum* subsp. *carotovorum* genome NC_012917 was used as a reference genome. Generated locally Collinear Blocks (LCBs) were analyzed to hunt for unique regions for Pectobacterium sp. Regions within the tyrR family transcriptional regulator gene were selected to design RPA primers and probe to specifically detect all *Pectobacterium* sp. RPA primers and probes were designed manually and checked for thermodynamic characteristics following the parameters described by Arif and Ochoa-Corona^28^. The tyrR gene location was represented using the BLAST Ring Image Generator (BRIGS)^29^; ncbi-blast 2.6.0+ database was used to compare and generate BRIG image. Average Nucleotide Identity (ANI) was calculated using Orthologous Average Nucleotide Identity Tool (OAT)^30^. The reverse primer PCRT-RPAR1 was labeled with biotin at 5′ position, and similarly, probe was labeled with FAM (Table 2). The Internal Transcribed Spacer (ITS) region was used to design the primers and probe to target host genomes of tomato and potato; primers and probe information are provided in Table 2. Primers and probes were synthesized by Integrated DNA Technologies Inc. (IDT, Coralville, IA) and Biosearch Technologies Inc. (Novato, CA).

**Table 2:** Detail of Recombinase Polymerase Amplification (RPA) primers and probes designed to detect genus *Pectobacterium* and host DNA.

Primer/Probe Name	Sequence	Length (nt)	GC%
PCRT-RPAF1	5′-CTGGATATGAAAGGGAAACCGGAGTTATTTAATCC-3′	35	40
PCRT-RPAR1	5′-/Biosg/GTCATTTCCAGCAAGAAATCCTGACCGCGAATCA-3′	35	46
PCRT-LP	5′-/FAM/TGTTTGAGCAATCGGCAGAGACCATCAGCG/Internal dSpacer/ATTTGACGATTGGCAGCA/Spacer/-3′	48	50
IC-RPAF1	5′-AACACAAACGACTCTCGGCAACGGATATCTCG-3′	32	50
IC-RPAR1	5′-/Biosg/ATGGCTTCGGGCGCAACTTGCGTTCAAAGACT-3′	32	53
IC-LP	5′-/FAM/TGAAGAACGTAGCGAAATGCGATACTTGGT/Internal dSpacer/TGAATTGCAGAATCCCGTGA/Spacer/-3′	50	44

### RPA assay

A 50 μl reaction was performed with the TwistAmp Exo® kit following the manufacturer’s protocols. Each reaction contained 29.5 μl of rehydration buffer, 0.6 μl of (10 μM) of probe, 2.1 μl (10 μM) of each forward and biotin-labeled reverse primer, 11.2 μl nuclease-free water, 2 μl of DNA template or plant sap, 2.5 μl of magnesium acetate to activate the RPA reaction. RPA reactions were performed at a constant temperature of 37 °C for 30 min. Each RPA run was conducted with positive control and a non-template control (NTC; water). After amplification, 2 μl of RPA product was added to a mix of 400 μl of nuclease-free water and 100 μl of buffer (Milenia Biotec). A Lateral Flow Device was vertically inserted into the dilution mix and left for 5 min.

### Bacterial-infected fruit materials and DNA extraction (host control)

Individual tomato fruit was mechanically inoculated in a biosafety hood with five bacterial strains of *Pectobacterium* sp., A5280, A5278, A5368, A2688, and A3048 using 10 µl of 10^8^ (CFU/ml) of each bacterial suspension. Individual potato tubers were inoculated with the *Pectobacterium* sp. strains A5278, A5368 and *Dickeya* sp. strains A5415, A5581, and A5582. All inoculated fruits and tubers were incubated for 24 h at 28 °C. Afterward, the DNA was extracted using the Wizard Genomic DNA Purification Kit following the manufacturer’s protocols. Each DNA sample was checked using RPA for *Pectobacterium* and host DNA.

### RPA sensitivity and spiked assays

Both, sensitivity and spiked assays were performed following the procedure mentioned by Larrea-Sarmiento *et al*.^31^ and Dobhal *et al*.^32^. DNA of *P*. *carotovorum* subsp. *carotovorum* strain A5280 was 10-fold serially diluted from 1 ng to 1 fg and used for sensitivity assays. The spiked assay was performed by adding 1 µl of host DNA (98 ng/µl) in each 10-fold serially diluted sensitivity reaction. NTC was included in each assay to confirm the reliability of the assay.

### RPA assay test with infected fruit samples without DNA prep

Individual tomato fruits and potato tubers were surface sterilized by immersing in a beaker containing 10% sodium hypochlorite for 3 min followed by three successive washes with sterilized water. Tomato fruits were mechanically inoculated in a biosafety hood (BSC-II) with *P*. *carotovorum* (strain A5278) using 10 µl of 10^8^ (CFU/ml) of bacterial suspension. Potato tubers were cut into thin slices using a sterilized knife. Cut potato slices were placed inside the Petri dishes containing filter paper with 1–2 ml of sterile water to make a moist chamber. Bacterial colonies from *P*. *carotovorum* (strain A5278) were stab inoculated into the middle of potato slice using a sterile tooth pick. Tomato fruits and tubers were incubated for 24 h at 28 °C. The target *P*. *carotovorum*, tomato, and potato were prepared using 20–50 mg infected fruit tissues and 400 µl (TE) buffer. Samples were macerated using an Eppendorf tube and pestle for 2 min. Tubes containing macerated tissues were left 10 min at room temperature to settle down the debris. Afterward, 2 μl supernatant was used in RPA reactions.

## Electronic supplementary material

Supplementary Dataset 1

## Data Availability

All sequencing data is available in NCBI GenBank database.
