# Supplementary material for: Genome-informed diagnostics for specific and rapid detection of Pectobacterium species using recombinase polymerase amplification coupled with a lateral flow device
Source: Sci Rep. 2018 Oct 29;8:15972. doi: 10.1038/s41598-018-34275-0 (PMC6206099; doi:10.1038/s41598-018-34275-0)
Supplement: Supplementary file 1 — Supplementary Dataset 1 [file 41598_2018_34275_MOESM1_ESM.pdf]

Genome-informed diagnostics for specific and rapid detection of *Pectobacterium* species using recombinase polymerase amplification coupled with a lateral flow device

Firas A. Ahmed[1,2], Adriana Larrea-Sarmiento[1], Anne M. Alvarez[1] & Mohammad Arif[1]*

[1]Department of Plant and Environmental Protection Sciences, University of Hawaii at Manoa, Honolulu, HI, United States; [2]Agriculture College, University of Kufa, Al-Najaf, Iraq

*Corresponding author: arif@hawaii.edu

Phone: 001-808-956-7765

Supplemental Table 1. Detail of primers used to amplify *dnaA* gene region of bacterial strains from genus *Clavibacter*, *Dickeya*, *Pectobacteriu*m, *Ralstonia*, and *Xanthomonas.*

Primer name	Forward primer (5'–3')	Reverse primer (5'–3')
Clav.dnaA	5-TACGGCTTCGACACCTTCG-3	5-CGGTGATCTTCTTGTTGGCG-3
Dic.dnaA	5-CACACYTATCGYTCCAAYGT-3	5-TGTCGTGACTTTCYTCRCGC-3
Pec.dnaA	5-ATGTGAACCCSAAACATACGT-3	5-TTCACGCAACTGCTCAATCTT-3
Ral.dnaA	5-TCRCGSCTGAACCSATCCT-3	5-TTGAGCTGSGCGTCCTTGC-3
Xan.dnaA	5-CAGCACGGTGGTGTGGTC-3	5-CCTGGATTCGCATTACACC-3
